# Functional Cortical Connectivity Related to Postural Control in Patients Six Weeks After Anterior Cruciate Ligament Reconstruction

**DOI:** 10.3389/fnhum.2021.655116

**Published:** 2021-07-15

**Authors:** Tim Lehmann, Daniel Büchel, Caroline Mouton, Alli Gokeler, Romain Seil, Jochen Baumeister

**Affiliations:** ^1^Exercise Science and Neuroscience Unit, Department of Exercise & Health, Faculty of Science, Paderborn University, Paderborn, Germany; ^2^Department of Orthopaedic Surgery, Clinique D’Eich, Centre Hospitalier de Luxembourg, Luxembourg, Luxembourg; ^3^Luxembourg Institute of Research in Orthopaedics, Sports Medicine and Science, Luxembourg, Luxembourg; ^4^Sports Medicine Research Laboratory, Luxembourg Institute of Health, Luxembourg, Luxembourg

**Keywords:** anterior crucial ligament reconstruction, postural control, functional neuroplasticity, compensatory mechanisms, functional connectivity

## Abstract

Whereas initial findings have already identified cortical patterns accompanying proprioceptive deficiencies in patients after anterior cruciate ligament reconstruction (ACLR), little is known about compensatory sensorimotor mechanisms for re-establishing postural control. Therefore, the aim of the present study was to explore leg dependent patterns of cortical contributions to postural control in patients 6 weeks following ACLR. A total of 12 patients after ACLR (25.1 ± 3.2 years, 178.1 ± 9.7 cm, 77.5 ± 14.4 kg) and another 12 gender, age, and activity matched healthy controls participated in this study. All subjects performed 10 × 30 s. single leg stances on each leg, equipped with 64-channel mobile electroencephalography (EEG). Postural stability was quantified by area of sway and sway velocity. Estimations of the weighted phase lag index were conducted as a cortical measure of functional connectivity. The findings showed significant group × leg interactions for increased functional connectivity in the anterior cruciate ligament (ACL) injured leg, predominantly including fronto−parietal [*F*_(1, 22)_ = 8.41, *p* ≤ 0.008, η^2^ = 0.28], fronto−occipital [*F*_(1, 22)_ = 4.43, *p* ≤ 0.047, η^2^ = 0.17], parieto−motor [*F*_(1, 22)_ = 10.30, *p* ≤ 0.004, η^2^ = 0.32], occipito−motor [*F*_(1, 22)_ = 5.21, *p* ≤ 0.032, η^2^ = 0.19], and occipito−parietal [*F*_(1, 22)_ = 4.60, *p* ≤ 0.043, η^2^ = 0.17] intra−hemispherical connections in the contralateral hemisphere and occipito−motor [*F*_(1, 22)_ = 7.33, *p* ≤ 0.013, η^2^ = 0.25] on the ipsilateral hemisphere to the injured leg. Higher functional connectivity in patients after ACLR, attained by increased emphasis of functional connections incorporating the somatosensory and visual areas, may serve as a compensatory mechanism to control postural stability of the injured leg in the early phase of rehabilitation. These preliminary results may help to develop new neurophysiological assessments for detecting functional deficiencies after ACLR in the future.

## Introduction

Injuries to the anterior cruciate ligament (ACL) substantially affect knee joint laxity and cause long-term consequences for injured athletes. The accompanying functional impairments of an ACL tear thereby appear to extend beyond biomechanical alterations, comprising a loss of mechanoreceptors which consequently lead to diminished afferent input to higher levels of the sensorimotor system (Courtney et al., 2005; Kapreli et al., 2009). Although ACL injuries have repeatedly been shown to cause deficits in knee function even after surgical anterior cruciate ligament reconstruction (ACLR), knowledge about associated mechanisms of the sensorimotor system for compensating these functional impairments is still lacking (Ageberg, 2002).

A growing amount of evidence has begun identifying clinically meaningful neuroplastic changes in the sensorimotor system following ACL injury. Investigations utilizing transcranial magnetic stimulation, for instance, have detected enhanced motor thresholds in the ACL injured limb, whereas functional magnetic resonance imaging or electroencephalography (EEG) studies observed increased activations of the motor areas and lower activations of somatosensory areas in these patients (Neto et al., 2019). Along with altered somatosensory information from the ACL, decreased innervation to the primary sensory cortex (Valeriani et al., 1999), as well as different corticospinal and motor cortex excitability (Pietrosimone et al., 2015; Grooms et al., 2017; Lepley et al., 2020) have been observed in patients after ACL reconstruction. As a consequence of increased motor thresholds of the injured limb and decreased responsiveness of motor areas, greater cortico-cortical stimulation is required to evoke efferent neural signaling in the motor cortex for properly controlling motion and stability of the knee joint (Lepley et al., 2020). Thus, patients with ACL injury have been shown to recruit motor areas to a larger extent than healthy individuals, indicating that cortical adaptations may facilitate the restoration of lower limb motor functions by driving compensatory synergistic muscle patterns (Courtney et al., 2005). Whereas initial findings have identified compensatory cortical patterns in patients after ACLR during proprioceptive tasks (Baumeister et al., 2008, 2011), little is known about the cortical mechanisms behind the postural deficiencies in this population.

After ACLR and the following rehabilitation, many patients exhibit significantly decreased static postural stability as implied by increased center of pressure (CoP) excursions and velocities while standing on their injured limb (Lehmann et al., 2017). Although comprehensive evidence in the early postsurgical period is missing, postural stability in patients after ACLR was reported to deviate from both the preoperative level (Gokalp et al., 2016) and healthy controls (Parus et al., 2015) after the 4th and 8th week of surgery. With respect to these functional deficiencies in patients after ACLR, postural control reflects multimodal interactions within the sensorimotor system (Shumway-Cook and Woollacott, 2012). Recent findings from neuroimaging studies suggested that active contributions from the cortex continuously maintain and restore postural equilibrium (Wittenberg et al., 2017). Collectively, these EEG investigations demonstrated variations in power spectral density of theta (4–7 Hz), alpha-1 (8–10 Hz), and alpha-2 (10–12 Hz) frequency oscillations in frontal, motor, parietal, and occipital regions of the cortex. While it is suggested that theta band oscillations reflect a general brain integrative mechanism related to short term storage and manipulation of multimodal information for a given operation, alpha oscillations are related to the active inhibition of non-essential neuronal processing (Cheron et al., 2016), with alpha-1 reflecting global alertness of cortical areas and alpha-2 being associated with task-specific sensorimotor processing (Pfurtscheller and Lopes, 1999). Modulations of oscillatory activity during postural tasks therefore conceivably reflect direct or indirect interactions within complex transcortical and cortico-subcortical loops for detecting and counteracting postural instability (Wittenberg et al., 2017). The underlying functional relationships, as quantified by statistical interdependencies among distributed cortical regions, are referred to as functional connectivity (Friston, 2011). These connections show frequency-specific modulations within a fronto-parietal theta network and a parieto-occipital alpha network in response to postural instability and varying postural demands (Mierau et al., 2017; Varghese et al., 2019; Lehmann et al., 2020).

In the light of injury-related increased postural sway (Lehmann et al., 2017), patients after ACLR may require stronger interactions of functionally interconnected sensorimotor areas for properly controlling postural stability (Rosen et al., 2019; Jiganti et al., 2020), as well as hip and knee movement (Criss et al., 2020) while standing on the injured limb. Investigations of structural white matter changes following ACLR further indicated that the hemisphere contralateral to the injured leg may be particularly affected by this neurostructural reorganization (Lepley et al., 2020).

Therefore, the aim of the present case-control study is to explore leg dependent patterns of cortical connectivity related to postural control during single leg stances in patients 6 weeks following ACLR. It is hypothesized that patients after ACLR may show compensatory cortical mechanisms in terms of stronger functional connections within the theta and alpha networks compared to their matched controls. Furthermore, these cortical adaptations may specifically affect the stance on the injured limb. In this way, the current investigation may gain further insight into sensorimotor changes related to postural deficiencies after ACLR.

## Materials and Methods

### Subjects

Twelve patients who underwent arthroscopic ACLR (seven left, five right) within the past 6–8 weeks were recruited to participate in the investigation (Table 1). In all cases, a semitendinosus/gracilis tendon autograft was used. Four patients also reported that concomitant meniscal repairs were performed. Furthermore, six patients had a history of previous ACL injury (three ipsilateral/three contralateral). Three of the patients underwent surgery for chronic ACL ruptures with a mean time of 441 ± 347 days between injury and surgery, whereas the remaining nine patients were operated for acute or subacute ruptures within a range of 42 ± 25 days. All patients actively participated in different sports (judo, soccer, fitness, team handball, athletics, and vaulting) prior to their injury. Collectively, patients declared their left leg as the preferred supporting leg when kicking a ball. Participants were excluded in cases of traumatic cartilage injuries, degenerative changes of the knee joint, chronic ankle instability or previous surgery to the ankle joint, as these may influence postural stability. With regards to the EEG measurements, medication intake of neuroactive or psychoactive drugs, implanted cardiac pacemaker, temporomandibular joint dysfunction, metal implants in the head or face, skull abnormalities or fractures, history of a neurological/psychological diseases, recurring or severe headaches/migraine, concussion within the past 6 months, previous heart or brain surgery, seizures at any time or history of epilepsy served as further exclusion criteria. Additionally, another sample of twelve healthy individuals served as a control group, mostly recruited from the same team or club. In this way, each patient was assigned a matched control based on sex (f/m), age (±3 years) and activity (same sport and sporting experience ±3 years). The control subjects were included following the same criteria as the ACLR group, except for any history of previous knee or ankle surgery. All participants were informed of relevant study details and gave written consent for their participation. The study was approved by the local research and ethics committee.

**TABLE 1:** Descriptive statistics for the anterior cruciate ligament reconstruction (ACLR) and control group.

	ACLR	Control	*t* Value	*p* Value
			
	(mean ± standard deviation)			
**Demographics**				
Sex (female/male)	5/7	5/7		
Age (years)	25.1 ± 3.2	25.5 ± 3.8	0.229	0.821
Height (cm)	178.1 ± 9.7	177.0 ± 9.6	−0.275	0.786
Weight (kg)	77.5 ± 14.4	73.7 ± 9.9	−0.743	0.465
Time injury to surgery (days)	142.1 ± 234.5	–		
Time post-surgery (days)	44.4 ± 4.5	–		
Sporting experience (years)	17.7 ± 4.8	16.6 ± 5.7	−0.541	0.594
Times active/week	4.5 ± 1.5	4.3 ± 1.7	−0.257	0.800
**KOOS score**				
Pain (%)	68.9 ± 15.2	98.6 ± 2.5	6.649	≤0.001*
Symptom (%)	56.2 ± 14.4	95.2 ± 3.8	9.030	≤0.001*
Activities of daily living (%)	78.6 ± 11.3	99.9 ± 0.4	6.499	≤0.001*
Sport/recreation (%)	20.4 ± 15.1	99.2 ± 2.9	17.694	≤0.001*
Quality of life (%)	39.6 ± 12.3	100.0 ± 0.0	17.003	≤0.001*
**IKDC score**				
Total score (%)	54.5 ± 8.3	99.8 ± 0.6	18.800	≤0.001*
Knee function (prior to injury)	9.6 ± 1.2	10.0 ± 0.0	1.239	0.228
Knee function (after injury)	4.7 ± 1.8	–		

KOOS, Knee Injury and Osteoarthritis Outcome Questionnaire; IKDC, International Knee Documentation Committee Questionnaire. *Significant difference (t-test) at p < 0.05.

### Experiment

Prior to the measurement, subjects were informed of the experimental procedures and an electrode cap was fitted to the head and a mobile EEG system was placed in a lightweight backpack. Afterward, subjects were asked to stand barefoot and center on a 600 mm × 400 mm triaxial force platform while keeping their arms relaxed alongside their body. Subjects performed one block of ten consecutive trials single leg standing on each supporting limb. The order of blocks (right leg–left leg/left leg–right leg) was randomized using a custom-built MATLAB (v.R2015b, Mathworks Inc., Natick, MA, United States) code. For excluding transition effects from bipedal to single leg stance, an auditory countdown was used to ensure that the subjects were standing in a stable single leg support position when the trials started. All trials lasted for 30 s with 15 s rest between trials. In order to limit confounding variables such as fatigue or pain, a 1-min break was implemented after the 5th trial of each block. Furthermore, subjects took a 5-min resting period between the two blocks. Thereby, the length of inter-trial breaks was adapted to the individual needs of the patients, if any of them requested a prolonged resting period. With respect to the stage of rehabilitation and functional recovery, no specific instructions for the stance and knee positions were given. Subjects were only instructed to stand on the supporting leg while holding the non-weight-bearing leg rear-facing in a comfortable flexion. For both single leg stances (right leg/left leg), subjects were asked to keep their gaze fixed on a 42 inches white flat screen four meters away at eye level to standardize the visual fixation point for all subjects and to prevent them from looking down at their feet.

### Patient Reported Outcomes

For the patient-reported measures of knee function, two corresponding scores were calculated.

The knee injury and osteoarthritis outcome questionnaire (KOOS; Roos et al., 1998) was used to evaluate the five dimensions: pain (9 items); symptoms (7 items); activities of daily life function (17 items); sport and recreation function (5 items); and knee-related quality of life (4 items). All items were scored from 0 to 4, and each of the five scores was calculated as the sum of items included. Additionally, the international knee documentation committee questionnaire (IKDC) was used to evaluate the three domains: symptoms (7 items), sports/daily activities (10 items) and current knee function (1 item), where high levels of symptoms or low levels of function scored 0 points. All item scores were summed and resulted in a total score (Collins et al., 2011). Furthermore, Visual Analog Scale (1–10) was used to track the perceived pain and exertion before and after each block of trials, scaling from “no pain” to “worst imaginable pain” (Haefeli and Elfering, 2006). No subject reported pain perception exceeding “mild” (2) symptoms.

### Postural Stability

Allocations of the CoP were collected by using a triaxial force plate (FP4060-05, Bertec, United States) and captured at a sampling rate of 1,000 Hz. The processing of the force plate data was conducted using a custom-built code in MATLAB (v.R2015b, Mathworks Inc., Natick, MA, United States). The code included removal of the first and last second of each trial, a fourth-order low-pass Butterworth filter with a cut-off frequency at 6.25 Hz, as well as detrending and downsampling (100 Hz) of the CoP data (Scoppa et al., 2013; Clark et al., 2014). Afterward, postural sway was quantified based on anteroposterior and mediolateral displacements of the CoP along the y-axis and x-axis, respectively, incorporating the CoP parameters area of sway and sway velocity (Lehmann et al., 2017). Sway velocity was calculated as the total distance tracked by the CoP per sample time, whereas the area of sway describes the ellipse area covered by 95% of the CoP trajectory in both anteroposterior and mediolateral direction within a trial.

### Cortical Activation

Cortical activity was continuously recorded from 65 active Ag/AgCl electrodes (actiCap, Brain Products, Munich, Germany) placed according to the extended international 10–20 system. EEG data was transmitted through a wireless transmission path (LiveAmp, Brain Products, Munich, Germany), digitally amplified at a sampling rate of 500 Hz and low-pass filtered online at 200 Hz. Electrode impedance at each electrode site was reduced to <5 kΩ in order to ensure an appropriate signal-to-noise ratio. All electrodes were referenced online to an FCz reference montage including AFz as the ground electrode. Additionally, a 3D electrode localization scanning system (BrainVision Captrak, Brain Products GmbH, Germany) was used to locate electrode positions on the scalp relative to anatomical landmarks placed on the nasion and preauricular points.

For processing of the EEG data, the EEGLAB toolbox (Delorme and Makeig, 2004) was used. Firstly, the individually digitized electrode information was transferred to the EEG recordings, including the circumference and shape of the head, as well as the Cartesian and polar coordinates for each electrode position (x, y, z, θ, φ, r). Consequently, the original reference channel was restored and added back to the data. Sinusoidal line noise (50/100 Hz) was removed (*CleanLine* Plugin)^1^ and a basic finite impulse response filter with a band pass of 3–30 Hz was applied. Finally, signal referencing was computed to common average and the data was offline downsampled to 256 Hz. In order to avoid influences on connectivity measures, *eBridge* (Alschuler et al., 2014) was used to identify channels that are linked by low-impedance electrical bridges. Additionally, noisy channel detection based on kurtosis, joint probability and spectrum of the recorded channel was performed using the EEGLAB *pop_rejchan* function. In case of channel rejections, a spherical spline interpolation of missing channels followed. After the preprocessing, non-stereotypical artifacts (e.g., electrode pops) were rejected through visual inspection. Further, stereotypical artifacts (eye blinks, muscle activity, and cardiac pulses) were removed by an adaptive mixture independent component analysis (Palmer et al., 2011) based artifact rejection. For this purpose, an automated independent component classifier (Pion-Tonachini et al., 2019) was utilized with respect to scalp topography, activity power spectrum and actual activity time course of identified sources. Ultimately, the remaining independent components were back-projected to the channel level.

Based on recommendations of Hardmeier et al. (2014), the clean data of concatenated trials was divided into 50 × 4-s epochs of 1,024 sample points per subject and standing leg, randomly selected from remaining 63 ± 3 cleaned epochs for the injured leg, as well as 64 ± 5 for the non-injured leg.

**FIGURE 1:**
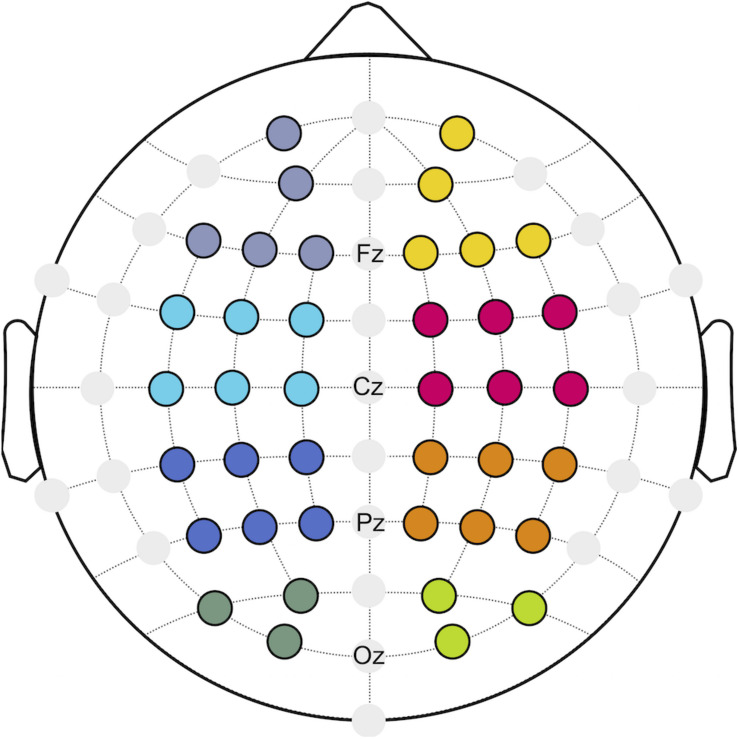
Regions of interest (ROIs) build for frontal (purple/yellow), motor (cyan/magenta), parietal (blue/orange), and occipital (dark green/green) areas.

With respect to neurostructural hemispherical differences and the importance of bilateral sensorimotor cortices for continuous postural control (Edwards et al., 2018), functional connectivity was investigated for the ipsilateral (I) and contralateral (C) hemisphere to the injured limb of patients after ACLR, as well as the corresponding matched leg of the control group. Referring to a previous investigation (Lehmann et al., 2020), eight regions of interest (ROIs; Figure 1) were created to describe the topology of connectivity across frontal (fI/fC: *AF3, F1, F5, F3, Fp1/F4, F6, F2, AF4, Fp2*), motor (mI/mC: *FC5, FC1, C3, FC3, C1, C5*/*C4, FC6, FC2, C6, C2, FC4*), parietal (pI/pC: *CP5, CP1, P3, CP3, P1, P5*/*P4, CP6, CP2, P6, P2, CP4*), and occipital areas (oI/oC: *O1, PO3, PO7/O2, PO4, PO8*).

For the quantification of functional connectivity, the weighted phase lag index (wPLI) was utilized to estimate functional connectivity among all possible pairs of channels between ROIs within the theta (4–7 Hz), alpha-1 (8–10 Hz), and alpha-2 (10–12 Hz) frequency bands by calculating the asymmetry of the instantaneous phase difference distribution between pairings of time series. As an extension of the traditional phase lag index, the wPLI is based on weighting each phase difference with respect to the imaginary component of the cross-spectrum (Vinck et al., 2011). It was assumed that the weighted phase lag between two signals is less sensitive to influences of common sources, reducing the probability of detecting false positive connectivity (Vinck et al., 2011).

### Statistical Analysis

Based on data from a similar study investigating cortical activity during single leg stances in patients after ACLR and healthy controls (Jiganti et al., 2020), a sample size calculation in G^∗^Power (Faul et al., 2007) indicated a minimum of 12 subjects per group to obtain a significant group × leg interaction for wPLIs at an alpha level of 0.05 and a power of 0.95.

The Statistical Package for Social Sciences (v.21, IBM, Chicago, IL, United States) was used for statistical analysis. Normal distribution of the demographics (except for time after injury and time post-surgery), CoP and EEG data was verified using the Shapiro–Wilks-test. Unpaired *t*-tests were applied to KOOS and IKDC data to determine statistical differences between groups. Since the preferred stance limb was equal for both groups, the injured and non-injured legs of the patients after ACLR were matched with the same-sided leg of their controls. In order to investigate patterns of postural stability and functional connectivity, a 2 × 2 [group by leg] mixed model analysis of variance (ANOVA) was calculated to determine the interaction effects for the corresponding measures. Additionally, repeated measures analyses of co-variance (rmANCOVA) were used to assess the effect of the side of injury on the dependent variables. The level of significance was set at *p* ≤ 0.05. Effect sizes were calculated using eta squared (η^2^) to assess the magnitude of differences between groups and leg, interpreting η^2^ ≥ 0.01 as small, η^2^ ≥ 0.06 as medium, and η^2^ ≥ 0.14 as large effects (Cohen, 1988). All measures considered for the mixed model ANOVA met the assumptions of a normal distribution (Shapiro–Wilk test), homogeneity of variance (Levene test) and sphericity (Mauchly test).

## Results

### Demographics and Subjective Knee Function

Demographic data and subjective knee function for both groups are shown in Table 1. No statistically significant differences between patients after ACLR and controls were found for age, height, weight, sport experience, or activity. The subjective rating of knee function, as assessed via KOOS and IKDC, demonstrated significantly lower knee function in patients after ACLR for all subscales. Overall knee function (IKDC) prior to the ACL injury showed no significant difference to the matched controls.

### Postural Sway

**FIGURE 2:**
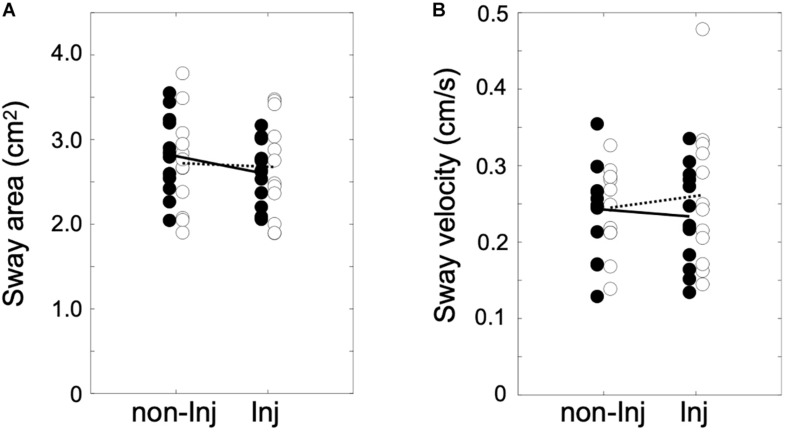
Area of sway in cm^2^ **(A)** and sway velocity in cm/s **(B)** for single leg stance on the injured (Inj) and non-injured leg (non-Inj) of the anterior cruciate ligament reconstruction (ACLR) group (white dots) and the control group (black dots) with their corresponding group means (ACLR, dotted line; control, solid line).

The analysis of postural sway (Figure 2) revealed no significant group × leg interaction for area of sway [*F*_(__1_, _22__)_ = 8.84, *p* ≤ 0.069, η^2^ = 0.14] or sway velocity [*F*_(__1_, _22__)_ = 2.36, *p* ≤ 0.084, η^2^ = 0.13].

### Functional Connectivity

**FIGURE 3:**
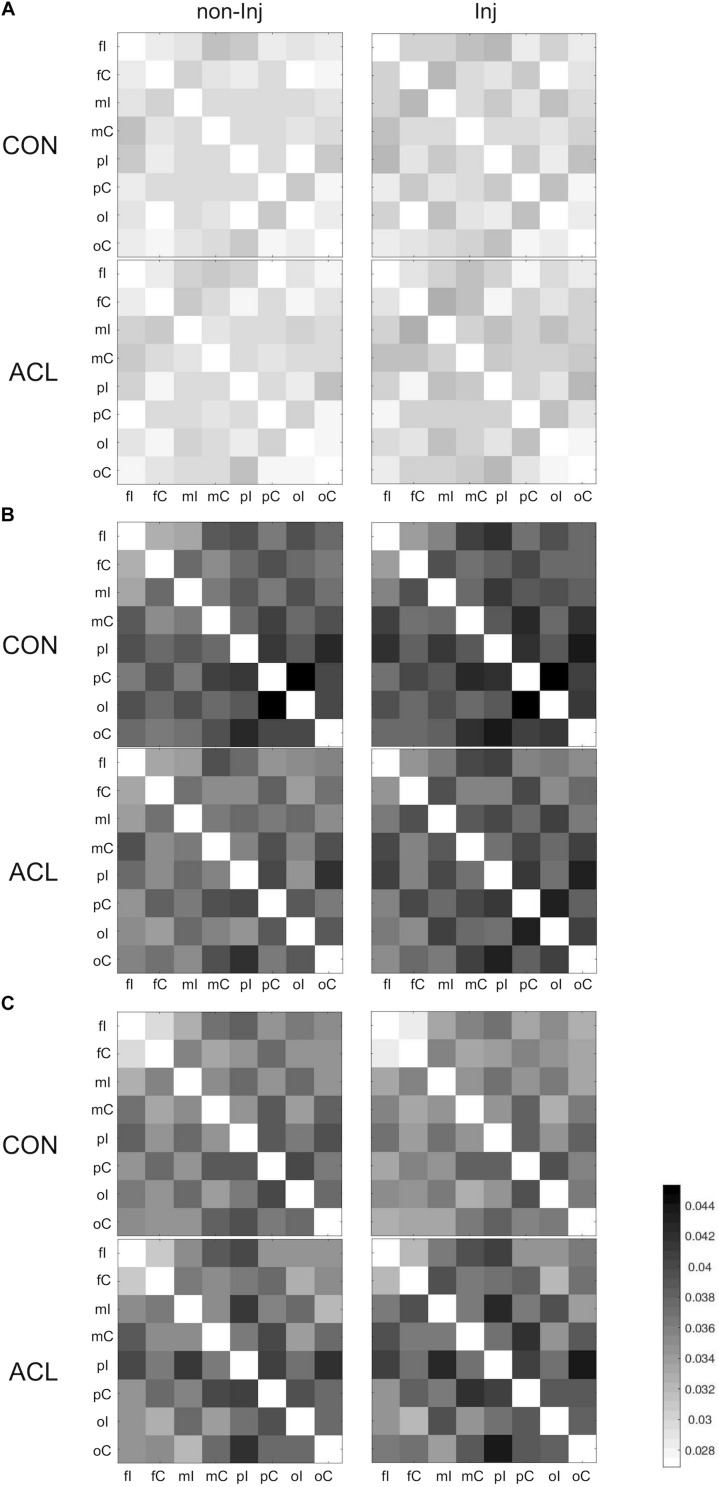
Weighted phase lag index (wPLI) matrices for all pairs of regions of interest (ROIs) during single leg stance on the non-injured (non-Inj, 1st column) and injured leg (Inj, 2nd column) for theta **(A)**, alpha-1 **(B)**, and alpha-2 **(C)**.

**FIGURE 4:**
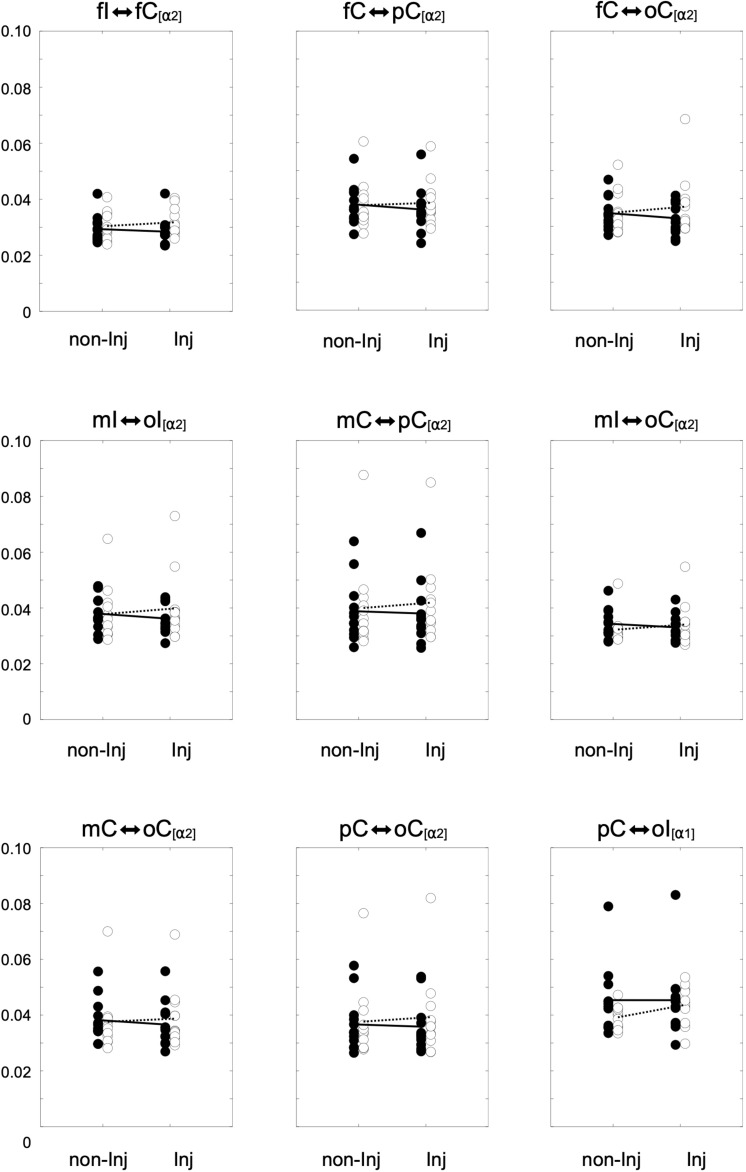
Distributions of the weighted phase lag index (wPLI) for significant group × leg interaction effects for the non-injured (non-Inj) and injured (Inj) stance leg in the alpha frequency band among ipsilateral frontal (fI), motor (mI), and occipital (oI) regions of interest (ROIs), as well as contralateral frontal (fC), motor (mC), parietal (pC), and occipital (oC) ROIs. Black points represent individual wPLIs for control subjects, white points for anterior cruciate ligament reconstruction (ACLR) patients. Trend lines indicate the corresponding condition change for controls (black line) and patients (dotted line).

Overall results for the wPLI within the different frequency bands are illustrated in Figure 3. The mixed ANOVA revealed no significant group × leg interaction for wPLIs in the theta band. In the alpha-1 frequency band, a significant group × leg interaction (Figure 4) was found for the connection between pC ⇔ oI [*F*_(__1_, _22__)_ = 6.64, *p* ≤ 0.017, η^2^ = 0.23]. For wPLIs within the alpha-2 frequency band, significant group × leg interactions (Figure 4) were found for fI ⇔ fC [*F*_(__1_, _22__)_ = 7.81, *p* ≤ 0.011, η^2^ = 0.26], fC ⇔ pC [*F*_(__1_, _22__)_ = 8.41, *p* ≤ 0.008, η^2^ = 0.28], mC ⇔ pC [*F*_(__1_, _22__)_ = 10.30, *p* ≤ 0.004, η^2^ = 0.32], mI ⇔ oI [*F*_(__1_, _22__)_ = 7.33, *p* ≤ 0.013, η^2^ = 0.25], fC ⇔ oC [*F*_(__1_, _22__)_ = 4.43, *p* ≤ 0.047, η^2^ = 0.17], mI ⇔ oC [*F*_(__1_, _22__)_ = 5.42, *p* ≤ 0.029, η^2^ = 0.20], mC ⇔ oC [*F*_(__1_, _22__)_ = 5.21, *p* ≤ 0.032, η^2^ = 0.19], and pC ⇔ oC [*F*_(__1_, _22__)_ = 4.60, *p* ≤ 0.043, η^2^ = 0.17]. In the ACLR group, wPLIs for these connections were greater when stance was performed with the non-injured leg than when stance was performed with the injured leg. In the control group, wPLIs for these connections were similar or slightly smaller when stance was performed with the matched non-injured leg than when stance was performed with the matched injured leg. The rmANCOVA model further revealed that the interaction between group and side of the injured leg had no significant influence on these dependent variables.

## Discussion

The present study explored cortical connectivity related to postural control in patients 6 weeks following ACL reconstruction. While patients after ACLR reported lower subjective knee function compared to their matched controls, postural sway revealed no significant differences in patterns between groups. Additionally, functional connectivity in the theta frequency band demonstrated similar characteristics in patients after ACLR and healthy controls in both stance legs. However, the alpha network characteristics differed as a function of stance and group. In patients after ACLR, alpha-2 connectivity was greater predominantly including connections within or linking with the contralateral hemisphere when standing on the injured leg, whereas the matched limbs of the controls exhibited a similar trend for both standing legs.

Parus et al. (2015) found postural deficiencies in patients after ACLR with medial meniscus suture 8 weeks post-surgery. In their study, patients demonstrated higher mean sway velocities in both the coronal and sagittal planes compared to the control group. In contrast to presumed assumptions of increased postural sway magnitude and velocity in patients after ACLR (Lehmann et al., 2017), no postural deficiencies were detected in the current investigation. As similarly observed in previous studies (O’Connell et al., 1998; Hoffman et al., 1999; Okuda et al., 2005; Jiganti et al., 2020), patients after ACLR did not show significantly different patterns in either sway area or sway velocity from their matched controls. Some evidence has already suggested that patients may adapt their sensorimotor control strategies after ACLR by increasingly involving visual information to maintain postural equilibrium. While these studies did not find a postural deficit in patients after ACLR under conditions with normal vision, patients demonstrated significantly decreased postural stability when vision was obstructed (O’Connell et al., 1998; Okuda et al., 2005). Therefore, the injured athletes in the present study may have developed early compensatory strategies to re-establish static postural stability through increasingly incorporating visual information to control static upright posture and knee stability.

**FIGURE 5:**
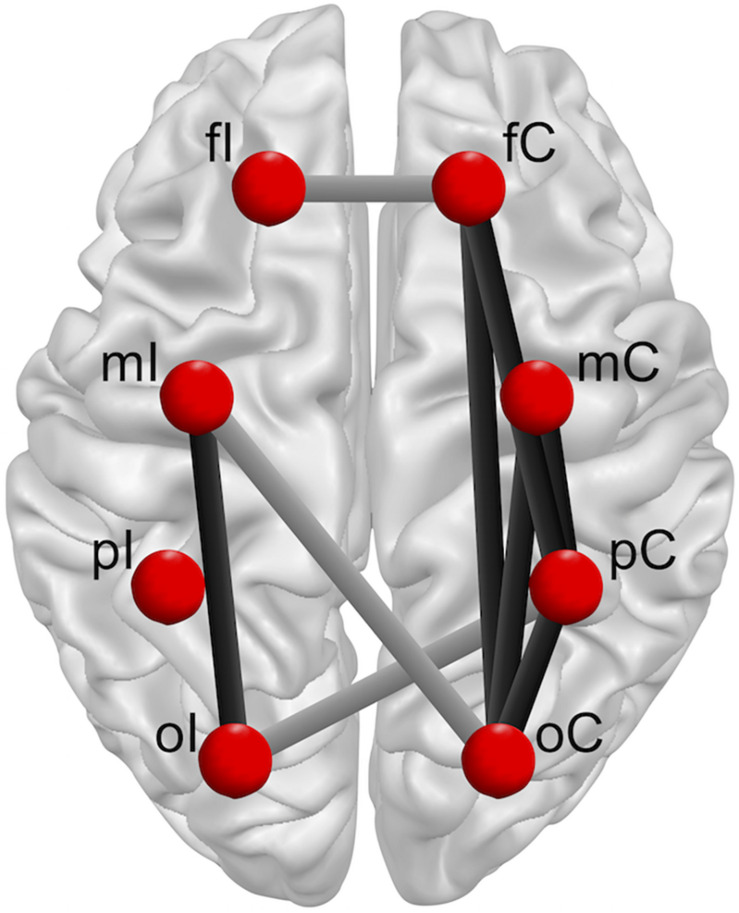
Overview group × leg interactions for intra-hemispherical (black line) and inter-hemispherical connections (gray line) between Regions of interest (ROIs) (red dots). Ipsilateral (fI, mI, pI, oI) and contralateral (fC, mC, pC, oC) ROIs are exemplary shown for single leg stance on the left injured leg. The brain model was illustrated with Brain Voyager Brain Tutor commercially available software (http://www.brainvoyager.com).

Nonetheless, in spite of similar sway scores in patients after ACLR and controls, the present findings indicate that inter-areal interactions during single leg stance may appear differently in the brains of patients after ACLR in comparison to their matched controls. Whereas multiple connections in the ACLR group showed stronger functional connectivity within the alpha frequency bands when standing on the injured versus the non-injured leg, controls demonstrated a similar pattern in both their matched limbs. Notably, this set of significantly modulated functional connections predominantly include fronto-parietal, fronto-occipital, occipito-motor, occipito-parietal, and parieto-occipital intra-hemispherical connections in the contralateral hemisphere, as well as an occipito-motor intra-hemispherical connection on the ipsilateral side (Figure 5). Furthermore, functional connectivity within inter-hemispherical connections of fronto-frontal, occipito-motor and occipito-parietal ROIs showed a different pattern comparing the stances between groups. All significant interactions also showed strong effect sizes (η^2^ > 0.14). Although postural control is traditionally linked to automatic processing originating from subcortical areas of the vestibulospinal, tectospinal and reticulospinal tract (Shumway-Cook and Woollacott, 2012), activation in frontal, motor, and somatosensory areas are assumed to actively govern these subcortical pathways in response to challenges in static postural stability (Wittenberg et al., 2017). Therefore, increased functional connectivity within a distributed sensorimotor network of fronto-parietal and occipito-motor areas may indicate higher demands on the postural control system in patients after ACLR when standing on their injured leg (Mierau et al., 2017; Varghese et al., 2019). Additionally, the present findings revealed that higher functional connectivity within the alpha-2 frequency band particularly appeared in the contralateral hemisphere to the injured standing leg of patients after ACLR. As proprioception and motor control of distal limbs is majorly ascribed to the contralateral cortical hemisphere (Shumway-Cook and Woollacott, 2012), it may be supposed that patients after ACLR require enhanced information processing to coordinate efferent neural signaling to maintain knee joint stability when standing on their injured limb (Pietrosimone et al., 2015; Grooms et al., 2017; Onate et al., 2019; Criss et al., 2020; Lepley et al., 2020). In fact, the current investigation demonstrated significantly different characteristics of occipito-motor and parieto-motor functional connections in ACLR. Generally, the somatosensory cortex in the parietal lobe, together with the visual cortex in the occipital lobe, are assigned a crucial role of integrating afferent sensory information into the sensorimotor system. Recent reports have further suggested that patients after ACLR may be especially reliant on visual information from the occipital cortex when performing postural tasks on their injured leg compared to healthy individuals (Okuda et al., 2005; Dingenen et al., 2015; Fernandes et al., 2016). Thus, higher functional connectivity of parieto-occipital and motor connections may point to stronger functional relationships and interactions between these related areas (Rubinov and Sporns, 2010). Based on this rationale, higher functional connectivity incorporating the somatosensory and visual areas may therefore serve as a compensatory mechanism to cope with the decreased responsiveness of motor areas in patients after ACLR (Onate et al., 2019; Criss et al., 2020; Lepley et al., 2020). Consequently, these elevated cortical contributions to maintain postural equilibrium could substantially affect more complex sensorimotor tasks, giving priority to postural stability at the expense of accuracy and speed of accompanying motor or cognitive actions (Mohammadi-Rad et al., 2016). In conclusion, investigating the functional reorganization of cortical networks in patients after ACLR may possess the potential to unveil underlying compensational mechanisms of the sensorimotor system beyond inconclusive mechanical or kinetic appearances (Needle et al., 2017), and to provide valuable knowledge for rehabilitative practice.

### Limitations

Nevertheless, some methodological limitations should be addressed. While most investigations of postural control in patients after ACLR solely included subjects with isolated and primary tears of the ACL, the experimental group in the present study consisted of a relatively heterogenous sample. Six patients reported a history of previous ACL injury and a total of four underwent concomitant meniscal repair in the course of their current surgical procedures. Similar to the ACL, menisci contain mechanoreceptors and are innervated by the posterior articular branch of the tibial nerve (Brindle et al., 2001). Consequently, meniscal tears have been reported to cause persistent proprioceptive deficits despite clinically successful surgical repairs (Al-Dadah et al., 2011). Whereas evidence is lacking regarding the effects of meniscal injury on sensorimotor processing, cortical contributions to postural control may appear differently in patients with concomitant meniscal tears in addition to ACL injury. Furthermore, the side of injury may constitute another limitation, as the ACLR sample in this study was comprised of both dominant and non-dominant leg injuries in patients. Although leg dominance may not have a significant impact on short-term functional outcomes following ACLR (Boo et al., 2020), a potential effect of leg dominance on postural stability could not be completely excluded (Promsri et al., 2020). While the affected side did not show significant effects on the group differences in functional connectivity, the unequal size of the subsamples generated for these analyses may have limited the statistical sensitivity. Based on the observations of the present study, further approaches may therefore explicitly investigate the influence of various factors such as concomitant injury, graft type or leg dominance on cortical processing related to postural control in patients after ACLR.

Apart from injury-related confounders, a potential explanation for failing to detect the expected postural deficiencies may be attributed to the preferred knee joint angle of the standing limb in the ACLR group. Based on visual observations, patients largely preferred a straight leg stance position with nearly full extension of their weight-bearing limb. As previously found, a straight-knee single leg stance paradigm may lack sensitivity to demonstrate decreased postural stability in patients after ACLR, whereas bent-knee single leg stances were able to distinguish postural performance of patients and controls (Kirsch et al., 2019).

Lastly, methodological limitations of the current investigation further refer to the technical procedures chosen for the analysis of cortical activity and functional connectivity. In this respect, it is important to note that channel recordings represent a linearly mixed multivariate signal of various sources of brain and non-brain signals (Lai et al., 2018). Channel data is therefore subject to volume conduction effects which could subsequently limit the probability of representing true functional connectivity of cortical areas. Although the wPLI minimized sensitivity to noise and volume conduction effects (Vinck et al., 2011), source space connectivity analysis may be even more suitable in depicting real phase synchrony between cortical areas (He et al., 2019). Hence, future studies are needed to develop more sophisticated and directed source modeling methods in order to provide further insights into cortical networks related to postural control in patients after ACLR.

## Conclusion

In summary, the present findings provided further insight into cortical activity related to postural control in patients 6 weeks following ACLR. While traditional measures of postural sway did not reveal altered patterns in the ACLR group of athletes in this timeframe, functional connectivity revealed differing patterns of cortical contributions to postural control. Moreover, increased functional connectivity was found irrespective of heterogenous patient characteristics such as sex, injured leg, or sporting activity. Based on the current findings, it may be speculated that patients require increased cortico-cortical connectivity to control postural stability of the injured leg in the early phases of rehabilitation after ACLR. In order to embrace future research to improve rehabilitation outcomes after ACL injury, these preliminary results may help develop new solutions for neurophysiological monitoring of functional deficiencies after ACLR. However, as the functional role of these network modulations remains uncertain, further studies utilizing sophisticated connectivity approaches in different homogenous subgroups of patients are needed to develop appropriate neurophysiological assessments for the monitoring of functional progress in patients after ACLR.

## Data Availability Statement

The datasets presented in this article are not readily available because of the local research data policy. Requests to access the datasets should be directed to tim.lehmann@uni-paderborn.de.

## Ethics Statement

The studies involving human participants were reviewed and approved by the Comité National d’Ethique de Recherche Luxembourg and the local Ethics Committee of Paderborn University. The patients/participants provided their written informed consent to participate in this study.

## Author Contributions

TL, DB, CM, AG, RS, and JB conceived and designed the experiments. The experimental procedures were conducted by TL and DB. TL analyzed the data and wrote the manuscript. The entire process was supervised by JB and RS. All authors read and approved the final manuscript.

## Conflict of Interest

The authors declare that the research was conducted in the absence of any commercial or financial relationships that could be construed as a potential conflict of interest.

## Footnotes

1 http://www.nitrc.org/projects/cleanline
